# A Mental Health Drop-In Centre Offering Brief Transdiagnostic Psychological Assessment and Treatment in a Paediatric Hospital Setting: A One-Year Descriptive Study

**DOI:** 10.3390/ijerph18105369

**Published:** 2021-05-18

**Authors:** Matteo Catanzano, Sophie D Bennett, Marc S Tibber, Anna E Coughtrey, Holan Liang, Isobel Heyman, The Lucy Project Team, Roz Shafran

**Affiliations:** 1 UCL Great Ormond Street Institute of Child Health, University College London, London WC1N 1EH, UK; sophie.bennett.10@ucl.ac.uk (S.D.B.); anna.coughtrey.10@ucl.ac.uk (A.E.C.); h.liang@ucl.ac.uk (H.L.); i.heyman@ucl.ac.uk (I.H.); PsychMedResearch@gosh.nhs.uk (T.L.P.T.); r.shafran@ucl.ac.uk (R.S.); 2 Great Ormond Street Hospital for Children NHS Foundation Trust, London WC1N 3JH, UK; 3 Research Department of Clinical, Educational and Health Psychology, University College London, London WC1E 6BT, UK; m.tibber@ucl.ac.uk

**Keywords:** mental health, cognitive-behavioral therapy, chronic illness, evidence-based practice, health care services

## Abstract

Aim: This study was part of a broader project to examine the acceptability, feasibility and impact of a transdiagnostic mental health drop-in centre offering brief psychological assessment and treatment for children and young people and/or their families with mental health needs in the context of long-term physical health conditions (LTCs). The aims of this investigation were to characterise: (i) the use of such a centre, (ii) the demographics and symptoms of those presenting to the centre, and (iii) the types of support that are requested and/or indicated. Methods: A mental health “booth” was located in reception of a national paediatric hospital over one year. Characteristics of young people with LTCs and their siblings/parents attending the booth were defined. Emotional/behavioural symptoms were measured using standardised questionnaires including the Strengths and Difficulties Questionnaire (SDQ). Participants subsequently received one of four categories of intervention: brief transdiagnostic cognitive behaviour therapy (CBT), referral to other services, neurodevelopmental assessment or signposting to resources. Results: One hundred and twenty-eight participants were recruited. The mean age of young people was 9.14 years (standard deviation: 4.28); 61% identified as white and 45% were male. Over half of young people recruited scored in the clinical range with respect to the SDQ. Presenting problems included: anxiety (49%), challenging behaviour (35%), low mood (22%) and other (15%). Conclusions: A considerable proportion of young people with LTC in a paediatric hospital scored in the clinical range for common mental health problems, indicating a potential for psychological interventions.

## 1. Introduction

Transdiagnostic interventions that cut across traditional diagnostic boundaries and promise to provide novel insights into how we might understand and treat mental health disorders have been gathering support [1]. These interventions apply the same underlying treatment principles across mental health disorders, without tailoring the protocol to specific diagnoses [2], though they still require a careful assessment and often incorporate disorder-specific strategies. Transdiagnostic clinical interventions have evolved in response to the “rampant” co-morbidity so often seen in both the community and clinical practice [1]. In a representative US population sample, 25.5% of children with a diagnosis had two or more diagnoses [3]. In clinical samples, diagnostic comorbidity rates are as high as 75% [4].

Pragmatic benefits of transdiagnostic interventions include the fact that practitioners can adopt universal approaches without modification for co-morbidity, sub-syndromal symptoms, complex formulations and other features that do not fit neatly within diagnostic boundaries. This should better enable goodness-of-fit between the intervention protocol and the individual patient, allowing protocol flexibility within fidelity [1]. There is also emerging evidence that transdiagnostic interventions may be cost-effective compared to treatment as usual [5]. Compared to disorder-specific protocols, training therapists in the delivery of one protocol targeting comorbid disorders may be more efficient and cost-effective. From a public health point of view this should make transdiagnostic interventions more scalable and increase their reach.

Young people with long term physical health conditions (LTCs; e.g., asthma, diabetes and epilepsy) are at a significantly greater risk of developing co-morbid emotional and behavioural difficulties than the general population, and so too are their families [6,7,8]. These difficulties not only distress the child and family and can impair friendships and school attendance, but can also negatively impact the management and course of the physical illness [9] and incur significant costs to health services. For example, between 12% and 18% of all National Health Service (NHS) expenditure on long-term conditions is linked to poor mental health, most commonly in the form depression and anxiety disorders [10]. In the USA, population studies have shown a trend of increased number, duration and cost of admission to children’s hospitals, if the patient has an additional psychiatric disorder [11].

The provision of paediatric psychology in the UK is typically to provide input when the problem is related to the LTC, for example difficulties adjusting to a diagnosis, needle phobia, problems disclosing the diagnosis to peers. Where there is a mental health problem that is not related to the LTC, for example depression or anxiety, a referral to local child and adolescent mental health services (CAMHS) is made. A review of access to CAMHS in the UK found that at least 4% of referrals explicitly mentioned mental health problems in the context of a LTC [12]. The same report suggests it is not known what happens to these referrals for the majority of these young people [12]. With mounting pressures on CAMHS, many services have developed elevated thresholds for accepting referrals and there is a tendency for children’s mental health needs to be overshadowed by their physical health diagnosis [13]. This suggests that young people with a LTC are not being seen by existing child mental health services and even if they are, due to long waiting lists, early intervention in the course of the mental health problem is not possible.

Once identified, mental health problems in young people with LTC respond to evidence-based psychological interventions [14]. In cases where siblings and parents also have mental health difficulties, these respond to evidence-based psychological interventions targeting parents or siblings [15,16]. Research to date suggests that whilst some adaptations to treatment may be needed, these are relatively minor and typically do not require significant specialist knowledge of paediatric medicine. For example, in working psychologically with young people with LTC it is helpful that sessions are offered flexibly to accommodate the location and times of medical appointments [17]. One way to accommodate families with these pressures may be to offer brief psychological interventions, which have been shown to be effective in young people with a LTC and elevated symptoms of anxiety [18]. Definitions of “brief psychological intervention” are varied, and distinct from “low-intensity” interventions, they usually involve fewer sessions (i.e., 50% or less therapy contact time than traditional “high-intensity” therapies that typically involve 12–16 sessions by a highly trained mental health professional), provided face-to-face or via video-facility by a mental health worker with a core professional qualification or equivalent (e.g., accredited CBT practitioners; usually individuals in the UK with a psychology or relevant degree who have undergone an additional year of training as part of the UK Increasing Access to Psychological Therapies or IAPT program) and are an abbreviated version of the full CBT intervention, supplemented with provision of between-session materials and exercises [19]. CBT refers to a psychological treatment integrating cognitive and behavioural approaches. In CBT the patient is helped to recognize unhelpful patterns of thinking and behaviour. Systematic discussion and carefully structured behavioural assignments are then used to help patients evaluate and modify both their thoughts and their behaviours. Some aspects of treatment have greater behavioural emphasis and others a greater cognitive emphasis [20]. Brief interventions have the potential advantage of reducing time spent travelling to clinic for patients, travel costs, time off work or school [21]. These benefits are likely to be particularly important to families attending a specialist paediatric hospital who already have to attend a number of appointments for their child’s physical illness.

One potential model of delivering brief psychological support to young people with a physical health condition is through the use of a mental health early identification, initial assessment, triage and brief transdiagnostic intervention drop-in centre located within the paediatric hospital. The rationale for the provision of brief interventions in this way includes the range of putative benefits for the young person as well as the service. For example, co-localisation of resources may facilitate efficient service integration and joint-work amongst professionals, as well as flexibility and ease of access for the young person. The early identification, initial assessment, triage and brief intervention model would enable support at the point of need with limited waiting times. This is particularly important given recent figures on national waiting times, with some children waiting up to 345 days from referral to the start of treatment [22]. Further, there is some evidence to suggest that self-referral services (of which this study is an example) improve accessibility for black and minority ethnic patients [23]. Moreover, this is in line with emerging international evidence, in Australia [24], Canada [25] and Ireland [26] that using brief psychological interventions to widen access to evidence-based treatments for mild-moderate mental health problems, can be an effective way to meet this rising demand. The little research into mental health “drop-in centres” for young people that has been completed demonstrated that such a model can be effective [27,28,29,30]. However, to the authors’ knowledge no research has explored their potential use in paediatric hospital settings.

This evaluation is part of a broader project to examine the acceptability, feasibility and impact of the Lucy Project: a “Mental Health and Psychological Wellbeing Drop-in Centre” in a tertiary paediatric hospital setting [31]. The Lucy Project was primarily designed to address common mental health problems such as anxiety, depression and challenging behaviour, for which a low intensity guided self-help intervention may be suitable [31]. The project was named after the character Lucy in the cartoon *Peanuts* who had a drop-in psychiatric clinic and was decided upon in consultation with children and young people at the hospital. The specific aims of this investigation were to characterise: (i) the use of an early identification, initial assessment, triage and brief intervention drop-in centre, (ii) the demographics and symptoms of those presenting to the centre, and (iii) the types of support that are requested and/or indicated.

Based on previous research we hypothesised that a minimum of 20% of young people with LTC and their siblings would present with mental health problems above the clinical threshold [32,33].

## 2. Methods

### 2.1. Design

This investigation was a cross sectional study of young people, their siblings and carers attending a national paediatric hospital.

### 2.2. Participants

For inclusion in the study, individuals had to have been a patient at the children’s hospital within the last six months, or be a carer/family member/sibling of such a patient. An additional inclusion criterion was a sufficient grasp of English to facilitate engagement with the assessment and treatment processes.

### 2.3. Ethics

Informed consent was taken for all participants included in the study. In the case of children under the age of 16, assent was obtained alongside parental consent.

### 2.4. Procedure

As this particular service did not exist prior to the research project, an initial piloting phase took place between January–February 2018 during which participants were recruited, having been made aware of the self-referral option primarily through leafletting. Leaflets were distributed in reception, waiting rooms, wards, the canteen, and other public spaces within the hospital. During recruitment sessions, volunteers and team members handed out leaflets to families in the reception and outpatient areas of the hospital. Project leaflets were also given out in the Physiotherapy service induction packs. This enabled piloting of procedures before purchase of the physical booth. Participants could refuse to take part in the study and still access treatment as usual (e.g., asking their paediatrician to refer them to paediatric psychology or asking their GP to refer them to local CAMHS). As this was a pilot feasibility and acceptability study, it was important to understand recruitment and retention rates, the types of presenting mental health problems, their severity and the proportion of participants allocated to various interventions.

### 2.5. The Booth

**Figure 1:**
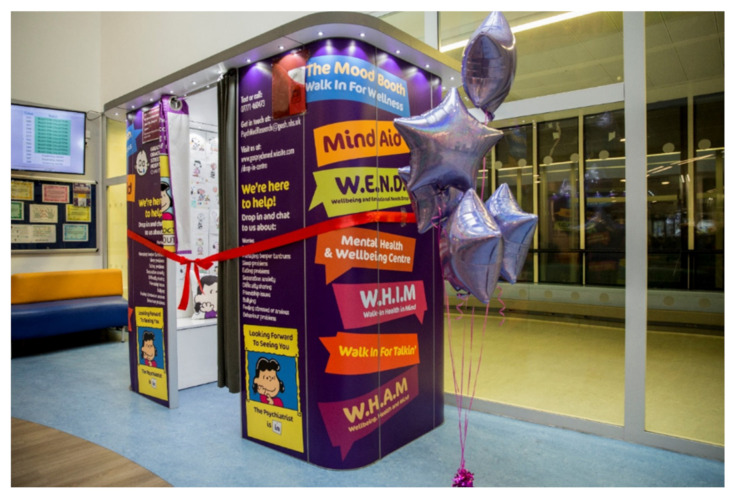
The booth.

The booth (see Figure 1) served both as a focus for recruitment and raising awareness of the project, as well as a physical space in which assessment and therapy sessions could be held. Additional therapy rooms were available within the hospital upon request. In practise, the booth was used primarily as a means of advertisement and a place for initial assessment where participants would complete the initial questionnaires, and time permitting, could be seen by a therapist for the initial assessment and/or brief intervention (e.g., signposting or a single session of psychoeducation). Follow-up appointments did not take place in the booth, but instead were either delivered by phone or in separate therapy rooms. The location of the booth was selected in order to maximise visibility and participant footfall. The booth (see Figure 1) was 1.59 × 1.23 × 2.03 metres (breadth, depth and height, respectively) and had contact information printed on its exterior as well as a list of symptoms and complaints that participants might want support with; these included “worries”, “feeling sad”, “managing temper tantrums”, “sleep problems”, “eating problems”, “separation anxiety”, “bullying”, “difficulty sharing”, “friendship issues”, “feeling stressed or anxious” and “behaviour problems”.

### 2.6. Recruitment

Recruitment took place from March–December 2018, with one volunteer/member of staff at the booth present Monday-Friday (10 a.m.–12 p.m. and 2 p.m.–4 p.m.). A clinical psychologist (working on the research study, but with an honorary contract with the hospital) and/or psychiatrist (part of the hospital staff, but working one day a week on the research project) was on call at all times.

All participants/families who were interested in taking part in the project were taken through the following stages before they could access treatment or other interventions: (i) provision of age-appropriate participant study information sheets for parent and young person (age ranges: 7–11, 12–15 and 16–18), (ii) taking of informed written consent, (iii) an initial assessment, and (iv) completion of child and parental baseline measures (parental measures were given to a parent even where the individual seeking support was the young person). To maximise flexibility and ease of engagement these stages could be completed immediately upon recruitment if the participant had time (i.e., at the booth itself), or else at a different time according to the individual’s needs, e.g., at subsequent face-to-face or phone-based appointments. In addition, baseline measures and consent forms could be completed by the participant in their own time and returned by email or stamped/return-addressed envelope provided by the researcher.

### 2.7. Intervention

#### 2.7.1. Early Identification

An important principle of the intervention involved early identification of people with mental health needs. Identification took place by five different routes: (i) the family/young person could approach a staff member at the booth (physical drop-in), (ii) the family/young person could contact the team by e-mail/telephone, (iii) a staff member could approach a family/young person in other areas of the hospital with a leaflet about the project (active recruitment), or (iv) clinicians within the hospital could signpost or (v) refer young people/families to the project. To facilitate the latter, members of the research team delivered presentations about the project to departments across the hospital. Six presentations were given in total to various departments within the hospital (Neurology, Ophthalmology, Rheumatology, Neurodisability, Genetics and Haematology/Oncology). These covered the background, aims and methods of the research, case studies of previous participants and a slide on how to contact the research team and/or signpost/refer patients. Approximately 10–30 professionals attended each presentation. Presentations were a method of advertising and promoting the project. In addition, consultants and head nurses of each department were informed of the project by email and leaflets about the project included within welcome packs in particular clinics.

#### 2.7.2. Initial Assessment

Once families had consented and completed baseline measures an initial assessment was carried out either over the telephone or face-to-face depending on participant preference. The therapists were able to offer same day assessments. Any delays between identification and assessment were due to participant preference or competing demands on their time. During the initial assessment the following information was systematically collected by the therapists using a standardised proforma (available upon request) designed specifically for the project: history of presenting problem (e.g., nature of anxiety/low mood symptoms, onset, triggers etc.), past medical history (e.g., diagnosis of epilepsy), family history, educational history, presence of active/past risk of harm to self (i.e., any history or current suicidal thoughts/thoughts of self-harm and/or suicide attempts/self-harm and/or risk of harm to others), presence of any neurodevelopmental diagnoses (e.g., autism spectrum disorder), past/current mental health assessment/treatment (e.g., previous psychological intervention by child and adolescent mental health services). Past/current mental health involvement was also cross-checked with the young person’s medical notes. This could detect past/current psychology involvement within the hospital or reference previous involvement in the community.

#### 2.7.3. Triage

Following the initial assessment, all participants were discussed in a triage meeting with a consultant child and adolescent psychiatrist who decided which intervention participants were allocated to. Given that the majority of the clinical work was being carried out by junior staff, it was considered important to have senior oversight over the clinical work and potential risk. This was provided by half a day per week of Consultant psychiatrist. Triage meetings took place every Thursday, so the longest time between assessment and triage would be seven days. If participants presented with considerable risk (e.g., current thoughts of self-harm/suicide or current self-harm), a risk assessment was completed and —where indicated—they were referred urgently to their local CAMHS and a risk management plan put in place. If the main presenting problem was post-traumatic stress disorder, possible psychosis, eating disorder or a severe emotional or behavioural problem (e.g., severe depression, anxiety or conduct disorder), a referral to CAMHS was made. If the presenting problem, whether for parent, child or sibling, was directly related to the physical health condition of the child (e.g., adjustment disorder/needle phobia/problems disclosing a medical diagnosis to peers) a referral was made to the paediatric psychologist attached to the relevant medical team within the hospital. If participants were actively receiving therapy from another therapist (e.g., having weekly sessions with a psychologist) the individual was signposted back to their therapist to avoid duplicating work.

In cases where the parent(s) presented with a mental health problem that was not related to their child’s physical health condition (e.g., anxiety and/or depression unrelated to the LTC) they were signposted to their local Increasing Access to Psychological Therapy (IAPT) service and if required assisted with the self-referral process. If the families wanted information/support groups or presented with mild problems and did not want/require a brief intervention they were signposted to resources (e.g., evidence-based self-help books or websites relevant to their child’s condition like the National Autistic Society). This was provided flexibly according to the family’s needs, either in person, by phone, by e-mailing or inclusion in a letter that was posted to the participant/family.

Where an undiagnosed neurodevelopmental condition (e.g., autism spectrum disorder or attention deficit hyperactivity disorder) was suspected during the initial assessment, following a discussion of the presenting symptoms with the Consultant psychiatrist in the triage meeting, participants would be offered the Development and Wellbeing Assessment (DAWBA) [34], which would be reviewed by the Consultant psychiatrist before deciding to offer a neurodevelopmental assessment. For young people presenting with anxiety, low mood and/or challenging behaviour a brief psychological intervention was offered. These categories were not mutually exclusive and participants could be allocated to more than one.

#### 2.7.4. Brief Intervention

Several outcomes were possible dependent on the outcome of the triage meeting. Thus, participants could: (i) be given/directed towards self-help materials and/or online resources, (ii) undergo a more extensive standardised diagnostic assessment using evidence-based assessment tools, e.g., the DAWBA, (iii) be sign-posted/referred to appropriate services, either internally or externally, (iv) see a therapist for brief CBT, defined as up to six sessions (6 h total) of either telephone or face-to-face guided self-help. Participants were offered an immediate brief intervention session and/or an appointment for a later date. In all cases the participant’s general practitioner was informed of their involvement in the study. Time between allocation and receiving the brief intervention varied depending on the intervention. Signposting to services or self-help materials or administration of the DAWBA were offered on the same day as allocation. Seeing a therapist for a brief intervention was often possible the same day and a first appointment was offered within seven days. Any delays were due to participant preference/competing demands on their time. More in-depth neurodevelopmental assessments were generally offered within 30 days. All of the interventions (onward referral, brief therapy, signposting and neurodevelopmental assessment for ADHD/ASD) were carried out by members of the research team (i.e., newly qualified clinical psychologists, trained psychological wellbeing practitioners (PWPs), consultant child and adolescent psychiatrist and/or a junior doctor with specific training in the intervention).

#### 2.7.5. Training and Supervision of Volunteers and Staff

Hospital volunteers were all already trained in safeguarding children, health and safety and equality and diversity as a routine part of their role. Additional training specific to the study was delivered by the Lucy Project team including clinical psychologists, PWPs and undergraduate student research assistants. The training consisted of reading a volunteer training handbook and online training including modules relating to: good clinical practice (GCP), research governance, informed consent and information governance. The volunteers were then observed during their first hour of recruitment by a study team member and subsequently supervised to recruit participants for two one-hour sessions (or until the supervisor considered them competent to recruit independently) before recruiting independently. Volunteers were supervised by the therapists on the project.

PWPs in the UK undergo one year of post-graduate training in carrying out brief assessments and delivering brief evidence-based interventions. As part of ongoing training and monitoring they received one hour, weekly, group supervision with a clinical psychologist and a separate one hour, weekly, triage meeting with a consultant child and adolescent psychiatrist. The therapists familiarised themselves with the brief interventions and were able to listen to tapes of therapists delivering these interventions as part of their training.

### 2.8. Measures

#### 2.8.1. Assessment of Interest

To measure interest in the project and uptake of the resource a number of indices of participant engagement were recorded. These included the average number and rate of participants recruited and the percentage of participants consenting who subsequently asked to be excluded or could not be contacted. In order to characterise relative use across different departments within the hospital, the nature of the participant’s current and past contact with psychological and medical services was recorded at the initial assessment phase as well as the department of current contact. This was subsequently cross-checked (with the participant’s consent) with medical notes on the hospital’s electronic records database.

#### 2.8.2. Participant Demographics, Symptom Profiles and Interventions Allocated to

All participants recruited completed a demographics questionnaire before the initial assessment, which recorded age, gender, ethnicity, post code, language status, and need for a translator. Post codes were converted to indices of multiple deprivation deciles using an online calculator (http://imd-by-postcode.opendatacommunities.org/imd/2019) (accessed on 4 December 2020). Indices of multiple deprivation have been developed in England to encompass material deprivation and aspects such as health, education and crime [35].

The main presenting symptom (e.g., anxiety/low mood/challenging behaviour/other) was noted at initial assessment by the therapist using the standardised proforma described above. Following triage, the interventions that participants were allocated to were noted and divided into one of four categories: brief CBT, referral, neurodevelopmental assessment or signposting to resources only.

#### 2.8.3. Child Mental Health Measures

Strengths and Difficulties Questionnaire (SDQ) [36,37]. A 25-item measure with robust psychometric properties, used to identify common emotional and behavioural symptoms (e.g., anxiety, low mood, conduct problems and hyperactivity) in children and young people. The SDQ has been shown to have moderate test-retest reliability [38], good concurrent [39] and discriminant validity [40]. In all cases, a parent-report form would be completed before the initial assessment and at six-months from baseline. Where the child was aged 11 or older, both parent and child (i.e., self-report) versions of the form were administered. For younger children (2–3 years of age) the 2–3 SDQ-parent version was used.

Children’s Global Assessment Scale [41]. A clinician rating of the child’s global functioning rated from 0 (very poor functioning) to 100 (very high functioning) with good concurrent validity and reliability between raters and across time [41]. This was completed after the initial assessment by the therapist who had conducted the assessment. Therapists were trained in administering the CGAS by an experienced child and adolescent psychiatrist. Initial CGAS scores were co-rated by both the therapists and the consultant psychiatrist in the triage meeting following initial assessment as part of training and they were considered trained once they had reached an acceptable inter-rater reliability. CGAS scores were subsequently rated independently by the therapist after the initial assessment.

#### 2.8.4. Parent Mental Health Measures (Self-Report)

Generalised Anxiety Disorder 7(GAD-7) [42]. A seven-item measure of the severity of generalised anxiety with good psychometric properties. The GAD-7 has been shown to have excellent internal consistency, good test-retest reliability, strong criterion validity [42] and evidence for construct, concurrent [43] and convergent [42] validity. Each item is rated on the options “not at all”, “several days”, “more than half the days”, and “nearly every day”. One parent/carer from every family would complete the GAD-7 before the initial assessment and at six-months from baseline. Scores ≥10 were considered to be above the clinical threshold.

Patient Health Questionnaire 9 (PHQ-9) [44]. A seven item measure of the severity depression with good psychometric properties. The PHQ-9 has been shown to have good internal consistency, good test-retest reliability and evidence for criterion validity [45]. Each item is rated on the options “not at all”, “several days”, “more than half the days”, and “nearly every day”. One parent/carer from every family would complete the PHQ-9 before the initial assessment and at six-months from baseline. Scores ≥10 were considered to be above the clinical threshold.

### 2.9. Analyses

Participant demographics and symptom profiles were compared to those of the wider hospital (requested via clinical information services), routinely collected national child and adolescent mental health service (CAMHS) outcome data from the Child Outcomes Research Consortium (CORC) dataset [46] and data from a national initiative to improve children’s access to evidence-based psychological therapies (CYP IAPT) [23,36,47] by running chi-square tests of homogeneity using R statistical software, version 3.6.3 (R Project for Statistical Computing). *Post hoc* analyses involving pairwise comparisons using multiple z-tests of two proportions with Bonferroni correction was applied where chi-square tests were statistically significant (*p* < 0.05). As the amount of clustered data was small, with only six families of those allocated to an intervention containing more than one participant, we accounted for clustering by removing those six families from the analysis. All descriptive statistics were undertaken using SPSS statistical analysis software (version 25, IBM).

## 3. Results

### 3.1. Early Identification

**Figure 2:**
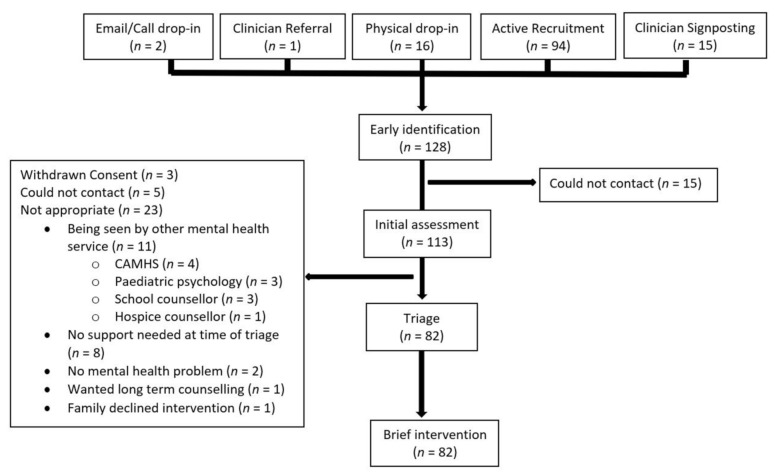
Flowchart showing the flow of participants through the various stages.

One thousand and thirty-seven families were provided with an information leaflet about the study as part of advertising the project to potential participants and 120 families consented to take part. Of these, 114 had one participant per family (87 patients, i.e., young people, attending the children’s hospital, four siblings and 23 parents), four had two participants per family (one patient-sibling, one patient-parent and two patient-patient dyads) and two had three participants per family (two patient-sibling-parent triads). No children came to seek the study without a carer/parent. Recruitment rates were steady throughout the period of the study, with approximately 10 participants recruited per month. Figure 2 illustrates the flow of participants through the study, with reasons for exclusion/attrition at each stage of the pathway. Participants were recruited from a broad cross-section of specialties/departments within the hospital, with the greatest number of participants recruited from Rheumatology and Neurology.

### 3.2. Initial Assessment

Fifteen participants could not be contacted between consent and initial assessment. This is because some participants would approach the booth on the way to an appointment. They would consent and give contact details and ask to be contacted and then not respond. Three participants withdrew consent at assessment. One said they were too busy; one did not give a reason and one stated they could not take part unless we could guarantee 100% confidentiality (i.e., even if risk was present).

### 3.3. Triage

Five could not be contacted to offer the intervention and therefore could not be allocated. As shown in Figure 2, 11 were already being seen by another mental health professional. Eight no longer needed support at the time of assessment. This happened because, for example, in one case, the participant had received positive news from the medical team. Two did not report a mental health problem at assessment, e.g., one participant was looking for help finding a nursery for their young child with a disability. One wanted long term counselling which we did not offer and was already on a waiting list for their local CAMHS. One teenager declined to engage with the assessment as they stated they would not engage with any intervention offered. Eighty-two participants were allocated to an intervention.

### 3.4. Brief Intervention

Of the 82 participants who completed the assessment and were allocated to an intervention (Table 1), 71 (87%) were children or adolescents (<18 years of age) and 11 (13%) were adults, of which 65 were young people with LTC who were patients at the hospital, six their siblings and 11 were parents. Gender, index of multiple deprivation deciles, ethnicity of participants and translation requirements were highly representative of patients presenting to the hospital more generally, though our sample was slightly older in age. However, when benchmarked against nationwide routinely collected CAMHS data from the CORC dataset, we saw a higher number of female, Asian and Black participants, and children were younger on average [47]. The majority of participants (59%) came from an area with an index of multiple deprivation decile between one and five and (92%) within <50 miles of London.

Symptom profiles are presented in Table 1. The primary problems for which participants sought support included: anxiety (49%), challenging behaviour (35%), low mood (22%) and other difficulties (15%); these included picky eating, sleeping problems, inattention, adjustment difficulties, requests for educational support and medically unexplained symptoms. These were similar to the national profile when benchmarked against UK CAMHS data, except for low mood, where rates were lower in our sample (*p* < 0.01). About half of participants had received no previous psychological or psychiatric assessment or treatment and only a minority (15%) had a history of risk. Rates of autism spectrum disorder and intellectual disability were similar when benchmarked against national CAMHS data.

Symptom severity scores are presented in Table 2. Over half of young people were in the clinical range for emotional and behavioural problems (i.e., scoring ≥ 16 if 2–3 years or≥ 17 if 4–17 years on the SDQ-p Total score [37]). The median score on the CGAS was in the range of ‘some noticeable problems’, suggesting mild to moderate degrees of functional impairment. The majority of young people had experienced emotional and behavioural problems for over a year (as measured by the SDQ-P). Over half (57%) of young people, scored above threshold on 2 or more subscales of the SDQ-P, suggesting high rates of co-morbidity. Approximately half of parents were in the clinical range for anxiety and/or depression (i.e., scoring ≥ 10 on the PHQ-9 and/or GAD-7).

Following triage, 31% of participants were provided a brief CBT intervention, 43% were referred internally to paediatric psychology, 7% were referred to local CAMHS, 12% underwent a neurodevelopmental assessment, 12% were referred for other services (e.g., other intervention research project for children with epilepsy and other local child mental health service research clinic) and 37% were signposted to resources/services. Note: some participants are represented more than once in these data since multiple outcomes/interventions were possible, e.g., neurodevelopmental assessment and subsequent onwards referral. In Table 1, a breakdown of the *primary* interventions to which participants were allocated is shown. 

## 4. Discussion

This study represents a preliminary evaluation of an early identification, initial assessment, triage and brief intervention drop-in centre in a tertiary paediatric hospital setting, which was designed to characterise the use, demographics, symptom presentation and interventions required of young people with LTC and/or their families seeking support in this context.

### 4.1. Early Identification and Initial Assessment

Participants recruited to the project broadly matched patients seen within the wider hospital with respect to gender, ethnicity and index of multiple deprivation decile. This suggests that the recruitment strategies employed here were successful in capturing a representative sample of the target population in this regard. With respect to ethnicity, participants from Asian and Black backgrounds were over-represented amongst participants recruited relative to nation-wide (CAMHS) data, but not hospital-wide data (Table 1). This suggests this model may be an effective way to increase access to mental health support amongst Asian and Black populations. Possible explanations may be the self-referral nature of the study, as there is evidence that the introduction of self-referrals to Improving Access to Psychological Therapies (IAPT) services may improve access for Asian and Black individuals to a level that is more reflective of the local population [23,48]. In addition, approaching individuals face-to-face with leaflets, as opposed to waiting for them to approach the project, i.e., “active recruitment”, may be a way to overcome some of the barriers to engagement that Asian and Black individuals face, such as consulting their GP for a referral, as individuals of Asian and Black ethnicity are less likely to consult their GP for mental health problems [49,50]. Furthermore, even when Asian and Black individuals do consult their GP, studies report that detection rates of mental health problems may be lower than for White British patients [51]. This is something that might have been overcome in the study by the routine use of measures such as the SDQ, which has been shown to be valid in ethnically diverse groups of young people [52]. Stigma around mental health, which may be amplified by particular cultural beliefs, may present another barrier to accessing treatment [53]. It is possible, therefore, that the location and visibility of the booth may have reduced some of this stigma.

A criticism sometimes raised against self-referral systems has been that they primarily increase access for “articulate middle-class clients” [54] and therefore cement inequality. However, the participants in the sample presented here came from areas with a broad spread of relative deprivation. Given that those in low-wage positions may not have the flexibility to attend several weekly appointments held during working hours [55], the study, which offered telephone appointments outside business hours, may have facilitated greater ease of access.

Another common concern with self-referral systems is that they will attract the “worried well” [56]. However, within the sample reported here we found not only that the majority of young people and parents were in the clinical range for common mental health disorders (e.g., anxiety and depression), but that most were in the clinical range for more than one mental health problem (e.g., emotional and conduct problems) and had experienced symptoms for an extended period (i.e., over one year as measured by the SDQ). Further, half had never accessed any mental health support previously. This suggests there is a proportion of young people and/or their families within the hospital who may be served by a service of the kind reported in this study.

### 4.2. Triage and Brief Intervention

Participants were triaged to a range of interventions. Over a third (43%) of the participants recruited to the project, were subsequently referred internally to existing psychological services within the hospital. This was either because they were already known to this psychologist (attached to their paediatric speciality) or because their mental health needs appeared to be directly related to their physical health condition (e.g., adjustment to illness, procedural fears etc.) This suggests that, in addition to providing brief psychological interventions within the hospital, the “booth” might act as a highly visible “single-point-of-access” for mental health care, guiding families to existing treatment pathways that they were previously unaware of.

A minority of participants (12%) were referred for further assessment. This is not surprising given that young people with genetic, neurological or other specific medical illnesses are at increased risk of neurodevelopmental disorders [57,58]. It may also reflect the sensitivity of systematic evidence-based assessment tools used as part of the study, e.g., the SDQ and DAWBA [59] and their potential to identify neurodevelopmental conditions. These neurodevelopmental difficulties might contribute to the presenting problem such as anxiety, school refusal, peer-difficulties, but not have been identified previously. These and the broader findings of the project highlight the potential value in systematic screening of young people for common psychological and neurodevelopmental conditions in a paediatric hospital, particularly where emotional and behavioural complaints are raised.

Limitations to this study include the small sample size and the preliminary nature of the evaluation. This cross-sectional analysis undertaken at a single time-point was designed to evaluate feasibility and accessibility, and no data are presented with respect to the impact of interventions provided, e.g., treatment outcome and participant satisfaction. However, this was not the remit of this particular study, and follow-up data have been presented in a separate publication [31]. We did not use validated measures of service use, as it was felt that questionnaire burden was already high. Future studies should look at service use more systematically, ideally as part of a comprehensive health economic evaluation.

## 5. Conclusions

A considerable proportion of young people with LTC and their family members accessing a transdiagnostic mental health early identification, initial assessment, triage and brief intervention drop-in centre in a tertiary level paediatric hospital, met the clinical threshold for two or more mental health problems (e.g., emotional and conduct problems). Despite evidence for chronicity of these mental health problems, few families appeared to have accessed appropriate effective services. Brief transdiagnostic interventions may be particularly well suited given this high level of co-morbidity.

We imagine there is a greater need, based on prevalence studies [32], than the number who consented into the study and that hospital-wide screening may be useful to further facilitate early identification [60]. Participants presenting to the study were representative of the wider hospital population and were more likely to be Black or Asian compared to CAMHS patients nationally, suggesting that this may be an effective and equitable method of improving access to psychological support, which may be particularly important from a public health point of view. A combination of onward referrals and immediate or planned transdiagnostic interventions were deemed to be appropriate in meeting the needs of the participants, suggesting a possible dual role for the drop-in centre as a single-point-of-access as well as a method for the delivery of brief transdiagnostic psychological interventions. Further research is required to evaluate the efficacy of such a resource and to determine the extent to which it can be transposed/translated to other settings.

## Figures and Tables

**Table 1:** Participant demographics. Core participant demographics are shown for all participants for whom data have been gathered along with the mean and standard deviation (SD), median and interquartile range (IQR), number (N) and percent (%) of cases (where relevant) for all data. IMD decile = Index of multiple deprivation decile.

		Drop-in Centre	Hospital-Wide Data	t/U/χ^2^/*z*; *p*	Nation-Wide Data	t/U/χ^2^/*z*; *p*
Age of <18s at the hospital in years, mean ± SD (range)		9.14 ± 4.28 (0–17)	6.8 ± 5.20 (0–17) ***	4.1; *p* < 0.001	11.64 ± 3.39 (0–17) ***	−6.8; *p* < 0.001
Age of parents, mean ± SD (range)		39.19 ± 8.57 (22–54)				
IMD decile, median (IQR)		5 (3–8)	5 (3–8)	1,615,771; *p* = 0.18		
Gender, % (n/n total)	Female	55 (45/82)	51	0.35; *p* = 0.55	47 (7565)	1.60; *p* = 0.21
	Male	45 (37/82)	49		53 (8440)	
Primary recipient of the intervention, % (n/n total)	Patient	80 (65/82)				
	Parent/carer	13 (11/82)				
	Sibling	7 (6/82)				-
Ethnicity, % (n/n total)	White	61 (43/81)	62	3.71; *p* = 0.45	82 ***	27.3; *p* < 0.001
	Asian	14 (11/81)	16		5 ***	11.9; *p* < 0.001
	Black	13 (10/81)	9		5 *	8.74; *p* = 0.016
	Any Mixed background	6 (5/81)	4		5	0.19; *p* = 1
	Any other ethnicity	6 (5/81)	9		3	3.86; *p* = 0.25
Parent relationship to child, % (n/n total)	Mother	87 (71/82)				
	Father	13 (11/82)				
Presenting problems, % (n/n total)	Anxiety	49 (40/82)			49	1.92; *p* = 0.66
	Challenging behaviour	35 (29/82)			34	1.78; *p* = 0.73
	Low mood	22 (18/82)			50 **	10.3; *p* = 0.005
	Other	15 (12/82)			14	0.50; *p* = 1
Co-morbidity defined as being above threshold on 2 or more SDQ-*p* subscales	Yes	57 (37/65)				
Known pre-existing neurodevelopmental diagnosis, % (n/n total)	ASD	9 (7/74)			8	0.06; *p* = 0.8
	Intellectual Disability	11 (8/74)			6	2.24; *p* = 0.14
	None	81 (60/74)				
Need for translator, % (n/n total)	Yes	7 (6/82)	4	1.6; *p* = 0.21		
	No	93 (76/82)	96			
County of origin, % (n/n total)	<50 miles of London	92 (68/74)				
	>50 miles of London	8 (6/74)				
History of mental health input, % (n/n total)	Yes	48 (38/80)				
	No	52 (42/80)				
History of risk present, % (n/n total)	Yes	15 (12/81)	-	-	-	-
Primary intervention allocated to, % (n/n total)	Low-intensity CBT	33 (27/82)				
	Referral	50 (41/82)				
	Neurodevelopmental assessment	6 (5/82)				
	Signposting to resources only	11 (9/82)				

Nationwide data from (Edbrooke-Childs et al., 2017) and (Wolpert et al., 2016); * *p* < 0.05; ** *p* < 0.01; *** *p* < 0.001.

**Table 2:** Participant symptoms. The median and interquartile range (IQR) for participants’ symptoms are presented for the parent report SDQ (2–3 years and 4–17 years) and GAD-7/PHQ-9 along with the corresponding clinical threshold and percentage of participants/parents above the clinical threshold. Clinical thresholds for the SDQ are taken from the newer four-band categorisation based on the parent completed version of the questionnaire.

		N/N Total	Median (IQR)	Missing *n* (%)		Clinical Threshold	% above Clinical Threshold
SDQ-Parent 4–17	Total	57/67	19 (13–26)	10 (15)	High	≥17	67
	Impact	55/67	4 (2–6)	12 (18)	Very high	≥2	80
	Length of difficulties	55/67	4 (4–4)	16 (22)			
	Burden	57/67	3 (2–3)	10 (15)			
	Emotional	57/67	6 (3–9)	10 (15)	High	≥5	63
	Conduct	57/67	3 (1–5)	10 (15)	High	≥4	49
	Hyperactivity	57/67	6 (3–8)	10 (15)	Slightly raised	≥8	35
	Peer	57/67	4 (2–6)	10 (15)	High	≥4	49
	Prosocial	57/67	7 (5–9)	10 (15)	High	≤6	49
SDQ-Parent 2–3	Total	8/8	16 (8–22)	0 (0)	High	≥16	50
	Impact	8/8	4 (1–6)	0 (0)	Very high	≥2	63
	Length of difficulties	8/8	4 (3–4)	0 (0)			
	Burden	8/8	3 (2–4)	0 (0)			
	Emotional	8/8	2 (1–6)	0 (0)	Close to average	≥4	38
	Conduct	8/8	4 (1–5)	0 (0)	Slightly raised	≥5	25
	Hyperactivity	8/8	7 (3–9)	0 (0)	High	≥7	50
	Peer	8/8	3 (1–4)	0 (0)	Slightly raised	≥4	50
	Prosocial	8/8	8 (5–10)	0 (0)	Close to average	≤5	25
PHQ-9		54/75	8 (3–15)	21 (28)	Moderate	≥10	44
GAD-7		54/75	10 (4–16)	21 (28)	Moderate	≥10	56
CGAS		66/75	60 (47–65)	9 (12)	Some noticeable problems		

Length of difficulties: Less than a month (score 1), 1–5 months (score 2), 6–12 months (score 3), Over a year (score 4) Burden (to others): Not at all (score 1), A little (score 2), a medium amount (score 3), a great deal (score 4).

## Data Availability

The data that support the findings of this study are available on reasonable request from the corresponding author. The data are not publicly available due to privacy or ethical restrictions.
